# Required propofol dose for anesthesia and time to emerge are affected by the use of antiepileptics: prospective cohort study

**DOI:** 10.1186/s12871-015-0006-z

**Published:** 2015-03-15

**Authors:** Kentaro Ouchi, Kazuna Sugiyama

**Affiliations:** Department of Dental Anesthesiology, Field of Oral and Maxillofacial Rehabilitation, Kagoshima University Graduate School of Medical and Dental Sciences, 8-35-1 Sakuragaoka, Kagoshima, Kagoshima 890-8544 Japan

**Keywords:** Neurological disorder, Dental treatment, Intravenous general anesthesia, Intellectual disability, Antiepileptic

## Abstract

**Background:**

We investigated the impact of the type of neurological disorder on the required propofol dose for anesthesia and the time to emerge from anesthesia during dental treatment in patients with autism (AU), cerebral palsy (CP), and intellectual disability (ID), some of whom also had epilepsy.

**Methods:**

We studied 224 patients with a neurological disorder who underwent dental treatment under intravenous general anesthesia. Patients were categorized according to neurological disorder (AU, CP, and ID; and with or without an antiepileptic). The propofol dose required for anesthesia, time to emerge, and modeled propofol blood concentration at emergence were evaluated.

**Results:**

In patients not given an antiepileptic, we found no significant differences in the propofol dose, modeled propofol blood concentration at emergence, or time to emerge among patients with AU, CP, and ID (P > 0.05). When using an antiepileptic, the dose of propofol (5.7 ± 1.51 mg/kg/h) was significantly lower than without an antiepileptic (6.8 ± 1.27 mg/kg/h) (P < 0.0001). The modeled propofol blood concentration at emergence in patients given an antiepileptic (0.5 ± 0.03 μg/ml) was significantly lower than without an antiepileptic (0.7 ± 0.02 μg/ml) (P < 0.0001). The time to emerge in patients given an antiepileptic (29.5 ± 12.5 min) was significantly longer than without an antiepileptic (21.6 min ± 10.0 min) (P < 0.0001).

**Conclusion:**

The propofol dose required for anesthesia and the time to emerge from anesthesia are not affected by the type of neurological disorder, but are affected by antiepileptic use.

**Trial registration:**

University Hospital Medical Information Network Clinical Trials Registry (UMIN000014179), Date of registration 4 June 2014.

## Background

Poor quality of oral health care in patients with neurological disorders has been recognized [1-3]. In dental practice, intravenous general anesthesia is useful for patients who are difficult to treat when not sedated such as those with neurological disorders [4]. Dental patients with intellectual disabilities need higher doses of sedatives than those without intellectual disabilities to obtain an adequate level of anesthesia [5]. Furthermore, one report has shown that autistic patients have low sensitivity to propofol compared to patients with intellectual disabilities [6]. Patients with cerebral palsy may need higher doses of sedatives due to decreased GABA_A_ receptor binding [7]. Neurological disorder patients often take an antiepileptic drug. Use of an antiepileptic drug may require a lower dose of propofol, because some types of antiepileptics are known to decrease hepatic metabolism [8].

Therefore, the objective of the present study was to investigate the relationship between the type of neurological disorder and the propofol dose required for anesthesia and the time to emerge from anesthesia during dental treatment in patients with autism, cerebral palsy, and intellectual disability. We also investigated these parameters in relation to use or non-use of an antiepileptic. This study used a prospective cohort study design.

## Methods

We studied dental patients with neurological disorders who were treated under intravenous general anesthesia at the Dentistry Outpatient Section for Patients with Neurological Disorders, Kagoshima University Medical Dental Hospital, from June 2007 to March 2013. Intravenous general anesthesia was administered by an anesthesiologist certified by the Japanese Board of Dental Anesthesiologists who had received the necessary training in general anesthesia. Written informed consent for dental treatment and this study under intravenous general anesthesia was obtained from each patient or from the guardian or caregiver in cases of patients with severe neurological disorders. The institutional review board of Kagoshima University Medical Dental Hospital approved the study protocol.

The definition of a neurological disorder was any patient who had been formally assessed and found to have autism, cerebral palsy, or intellectual disability. We excluded patients with cerebral palsy from the category of intellectual disability. Patients who did not use target-controlled infusion (TCI) or did not consent to the study were excluded.

### Intravenous general anesthesia protocol

**Figure 1:**
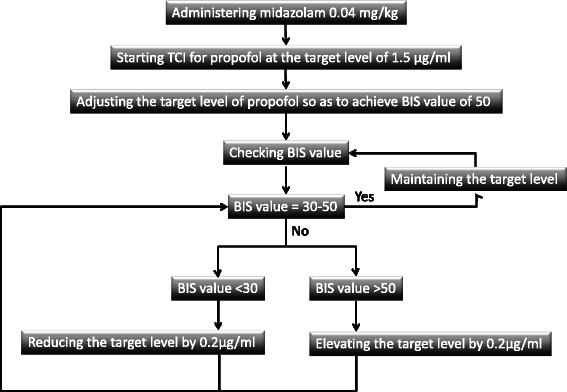
**Intravenous general anesthesia protocol.** TCI: target-controlled infusion. BIS: Bispectral index.

The patients did not receive premedication. A bispectral index sensor (BIS) (BIS Quatro, XP Platform; Aspect Medical Systems Inc., Norwood, MA, USA) was attached to the patient’s forehead and connected to a BIS monitor (A2000 BIS monitor XP, Aspect Medical Systems Inc.) to evaluate the level of intravenous general anesthesia. The BIS value was collected continuously and recorded every 15 s. Intravenous general anesthesia was induced with bolus intravenous administration of midazolam 0.04 mg kg^−1^ and maintained with continuous infusion of propofol. After the intravenous administration of midazolam, using a propofol TCI pump (3500 TCI, Graseby Medical Ltd., Hertfordshire, UK, or TERUFUSION TE-371, Terumo Co., Tokyo, Japan) with a built-in TCI system (Diprifusor, AstraZeneca Plc., London, UK) and according to the parameters reported by Marsh [9], continuous intravenous infusion of propofol was initiated using the TCI method. The dose of propofol was titrated to achieve a BIS of 50 and achieve an adequate level of anesthesia: asleep but not responding to stimulation. Dental treatment was initiated after the BIS value had stabilized. During dental treatment, the BIS value was maintained at 30–50 by adjusting the target propofol level using TCI. A local anesthetic was used appropriately by the operating dentist. Endotracheal intubation was not performed, and spontaneous breathing was maintained. Propofol was discontinued at the end of the dental treatment. The intravenous general anesthesia protocol for adjusting the propofol level is shown in Figure 1.

### Measurement of parameters

We investigated the dose of propofol administered, the time to emerge from intravenous general anesthesia, and the modeled propofol blood concentration at emergence. The dose of propofol administered (mg/kg/h) was defined as the required dose of propofol (mg)/patient’s body weight (kg)/administration time (h). Time to emerge (min) was defined as the time from the discontinuation of propofol until spontaneous eye opening of the patient with the patient’s name called every 3 min. The modeled propofol blood concentration at emergence (μg/ml) was defined as the value that was displayed in the plasma concentration site by the TCI pump when the patient awoke.

### Statistics

Two hundred twenty-four patients were included and categorized according to disability (autism, cerebral palsy, or intellectual disability; with or without an antiepileptic). We tested the data for normality with Levene's test. For comparison of more than three groups, a two-way ANOVA and the Tukey-Kramer test were employed for continuous variables. For comparison of two groups, the unpaired t-test was employed for continuous variables, and the chi-square test for categorical variables. JMP software (SAS Institute Inc., Japan) was used for statistical analysis, and P < 0.05 was regarded as statistically significant. The results are presented as the mean ± standard deviation (SD).

## Results

Tables 1 and 2 show the patients’ demographics. Two hundred twenty-four patients with neurological disorders were categorized into those with autism (AU; n = 62), cerebral palsy (CP; n = 28), or intellectual disability (ID; n = 134). Among these patients, 77 also had epilepsy and received antiepileptic medications, including carbamazepine, phenytoin, phenobarbital, zonisamide, topiramate, and valproate. Table 3 shows the number of patients receiving each type of antiepileptic. Among patients receiving antiepileptics, 65 patients received more than one antiepileptic. A local anesthetic (1:80,000 adrenaline including 2% lidocaine) was used appropriately during dental treatment, and consumption was less than 1.8 ml.

**Table 1:** **Patient demographics according to the type of disability**

	AU	CP	ID	P-value
Gender (M/F)	56/6	25/3	61/73	<0.0001
Age (years)	20.1 ± 7.92	27.5 ± 5.75	26.8 ± 5.97	<0.0001
Duration of dental procedure (min)	50.8 ± 17.93	47.7 ± 13.41	50 ± 16.73	0.72

**Table 2:** **Patient demographics according to whether an antiepileptic was used**

	No antiepileptic	Antiepileptic	P-value
Gender (M/F)	98/49	44/33	0.19
Age (years)	24.5 ± 8.04	26 ± 5.15	0.13
Duration of dental procedure (min)	49.2 ± 17.64	51.4 ± 14.61	0.34

**Table 3:** **Number of patients receiving each type of antiepileptic**

Antiepileptic	Number of patients receiving antiepileptic
Carbamazepine	51
Phenytoin	23
Phenobarbital	10
Zonisamide	14
Topiramate	21
Valproate	23

**Figure 2:**
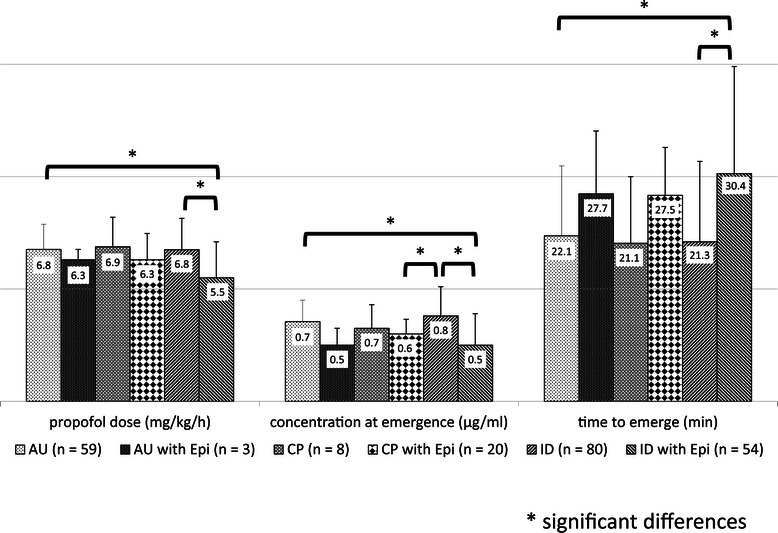
**Dose of propofol administered, time to emerge from anesthesia, and modeled propofol blood concentration at emergence in patients that used and did not use an antiepileptic for each neurological disorder.** The dose of propofol administered to patients who also received an antiepileptic was lower; the differences were significant between AU and ID with Epi, and between ID and ID with Epi. The modeled propofol blood concentration at emergence in patients given an antiepileptic was lower; the differences were significant between AU and ID with Epi, between ID and ID with Epi, and between ID and CP with Epi. The time to emerge from anesthesia in patients given an antiepileptic was longer; the differences were significant between AU and ID with Epi, and between ID and ID with Epi.

We compared the use and non-use of an antiepileptic in each category of neurological disorder. Patients were categorized into those with autism without an epileptic (AU; n = 59), autism with an epileptic (AU with Epi; n = 3), cerebral palsy without an epileptic (CP; n = 8), cerebral palsy with an epileptic (CP with Epi; n = 20), intellectual disability without an epileptic (ID; n = 80), and intellectual disability with an epileptic (ID with Epi; n = 54). We found significant differences in the dose of propofol administered (P < 0.0001), modeled propofol blood concentration at emergence (P < 0.0001), and time to emerge from anesthesia (P < 0.0001) among the six groups (Figure 2).

As a post-test, in patients not given an antiepileptic, we found no significant differences in the dose of propofol administered (mean ± SD; AU: 6.8 ± 1.12 mg/kg/h; CP: 6.9 ± 1.31 mg/kg/h; ID: 6.8 ± 1.39 mg/kg/h; P = 0.96), modeled propofol blood concentration at emergence (AU: 0.7 ± 0.19 μg/ml; CP: 0.7 ± 0.21 μg/ml; ID: 0.8 ± 0.26 μg/ml; P = 0.24), or time to emerge from anesthesia (AU: 22.1 ± 9.33 min; CP: 21.1 ± 8.90 min; ID: 21.3 ± 10.73 min; P = 0.90) among patients with AU (n = 59), CP (n = 8), and ID (n = 80) (Table 4).

**Table 4:** **Dose of propofol administered, modeled propofol blood concentration at emergence, and time to emerge from anesthesia, among the types of disabilities in patients not given an antiepileptic**

	AU	CP	ID	P-value
Dose of propofol (mg/kg/h)	6.8 ± 1.12	6.9 ± 1.31	6.8 ± 1.39	0.96
Concentration at emergence (μg/ml)	0.7 ± 0.19	0.7 ± 0.21	0.8 ± 0.26	0.24
Time to emerge (min)	22.1 ± 9.33	21.1 ± 8.90	21.3 ± 10.73	0.90

For the next post-test, we compared the use and non-use of an antiepileptic in each category of neurological disorder. The dose of propofol administered to patients who also received an antiepileptic was lower than that in patients not given an antiepileptic; the difference was significant between ID (6.8 ± 1.39 mg/kg/h) and ID with Epi (5.5 ± 1.60 mg/kg/h) (P < 0.0001). The modeled propofol blood concentration at emergence in patients given an antiepileptic was lower than in those not given an antiepileptic; the difference was significant between ID (0.8 ± 0.26 μg/ml) and ID with Epi (0.5 ± 0.28 μg/ml) (P < 0.0001). The time to emerge from anesthesia in patients given an antiepileptic was longer than in patients not given an antiepileptic for each neurological disorder; the difference was significant between ID (21.3 ± 10.73 min) and ID with Epi (30.4 ± 14.30 min) (P < 0.0001) (Table 5).

**Table 5:** **Dose of propofol administered, modeled propofol blood concentration at emergence, and time to emerge from anesthesia in patients that used and did not use an antiepileptic, for each type of disability**

		No antiepileptic	Antiepileptic	P-value
Dose of propofol (mg/kg/h)	AU	6.8 ± 1.12	6.3 ± 0.46	0.99
	CP	6.9 ± 1.31	6.3 ± 1.17	0.90
	ID	6.8 ± 1.39	5.5 ± 1.60	<0.0001
Concentration at emergence (μg/ml)	AU	0.7 ± 0.19	0.5 ± 0.15	0.82
	CP	0.7 ± 0.21	0.6 ± 0.13	0.96
	ID	0.8 ± 0.26	0.5 ± 0.28	<0.0001
Time to emerge (min)	AU	22.1 ± 9.33	27.7 ± 8.39	0.96
	CP	21.1 ± 8.90	27.5 ± 6.39	0.74
	ID	21.3 ± 10.73	30.4 ± 14.30	<0.0001

We next compared the use and non-use of an antiepileptic in all patients. The dose of propofol administered to patients who also received an antiepileptic (mean ± SD; 5.7 ± 1.51 μg/ml; n = 77) was significantly lower than that in patients not given an antiepileptic (6.8 ± 1.27 μg/ml; n = 147) (P < 0.0001). The modeled propofol blood concentration at emergence in patients given an antiepileptic (0.5 ± 0.03 μg/ml) was significantly lower than in those not given an antiepileptic (0.7 ± 0.02 μg/ml) (P < 0.0001). The time to emerge from anesthesia in patients given an antiepileptic (29.5 ± 12.5 min) was significantly longer than in patients not given an antiepileptic (21.6 ± 10.0 min) (P < 0.0001) (Table 6).

**Table 6:** **Dose of propofol administered, modeled propofol blood concentration at emergence, and time to emerge from anesthesia in patients that used and did not use an antiepileptic in all types of disabilities**

	No antiepileptic	Antiepileptic	P-value
Dose of propofol (mg/kg/h)	6.8 ± 1.27	5.7 ± 1.51	<0.0001
Concentration at emergence (μg/ml)	0.7 ± 0.02	0.5 ± 0.03	<0.0001
Time to emerge (min)	21.6 ± 10.0	29.5 ± 12.5	<0.0001

## Discussion

The objective of the present study was to investigate the relationship between the type of neurological disorder and the propofol dose required for anesthesia and time to emerge from anesthesia during dental treatment. In patients not given an antiepileptic, we found no differences in the required dose of propofol among patients with autism, cerebral palsy, and intellectual disability. The results of this study show that evaluation of sensitivity to anesthetics is necessary when considering the use of an antiepileptic, because the required dose of propofol in patients with ID given an antiepileptic was significantly lower than in those not given an antiepileptic.

Antiepileptic agents have the potential to inhibit drug metabolism, resulting in a number of interactions involving elevation of plasma concentrations of concomitantly administered drugs [10]. Most commonly used antiepileptics are eliminated via hepatic metabolism. Hepatic enzyme inhibition usually occurs because of competition at the enzyme site and results in a decrease in the rate of metabolism of the affected drug [10,11]. Clinically, this is associated with an increased plasma concentration of the affected drug and potentially an increased pharmacologic response. Metabolic reactions are catalyzed by cytochrome P450 (CYP) and uridine diphosphate glucosyltransferase (UGT) enzymes. CYP2B6, CYP2C9, and CYP2C19 contribute to the metabolism of propofol [12-14]. CYP2B6 contributes to the metabolism of valproate [15]. CYP2C9 contributes to the metabolism of carbamazepine, phenytoin, phenobarbital, and valproate. Thus, antiepileptics such as carbamazepine, phenytoin, phenobarbital, and valproate contribute to the competitive inhibition of hepatic CYP2B6 and CYP2C9, because metabolism CYP is the same as propofol. Zonisamide inhibits the propofol metabolizing enzymes CYP2C9 and CYP2C19 in vitro [16]. Valproate inhibits CYP2C9 in vitro [17]. Clinically, phenytoin inhibits CYP2C9, and similarly, topiramate inhibits CYP2C19 [18,19]. Carbamazepine inhibits 2C19 [20]. In addition, in vitro, phenytoin, phenobarbital, and valproate inhibit UGT 1A9, which mediates glucuronic acid conjugation, the main metabolic pathway of propofol [21-23]. In this way, all antiepileptics used by our study patients have an inhibitory effect on propofol metabolism. Thus, antiepileptic drugs decrease the clearance of propofol. Propofol may also be metabolized by non-liver mechanisms such as pulmonary and renal metabolism [24,25]. Inhibition of pulmonary metabolism and renal metabolism by antiepileptics has been not reported. Thus, antiepileptic drugs have been suggested to increase the blood concentration of propofol by inhibiting the action of CYP and UGT. Similarly, the metabolism of anesthetics such as propofol may be inhibited because different drugs competitively inhibit a common CYP [26,27]. Therefore, antiepileptic drugs reduce the required dose of propofol and extend the time needed for emergence from anesthesia.

Patients with ID have been reported to require higher doses of sedatives to obtain an adequate level of anesthesia [5]. Among patients with disabilities, those with AU require higher doses of sedatives to obtain an adequate level of anesthesia compared to patients with other disabilities such as ID [6]. In these reports, the group requiring a lower dose included those who were given an antiepileptic. Therefore, these reports suggest that patients with a neurological disorder or with ID require less propofol than patients not given an antiepileptic including those without a neurological disorder or with AU, due to the inhibitory action of the antiepileptic drug on CYP. Also, these reports suggest that the required dose of propofol may not be affected by the type of disability when excluding those given an antiepileptic.

Variations in individuals regarding the propofol dose required for anesthesia and the time for emergence from anesthesia are thought to result from pharmacokinetic and pharmacodynamic factors. The blood concentration of propofol may increase in proportion to the administered dose if drug metabolism is slow. Assessment of the anesthesia level according to an objective parameter such as an exclusive electroencephalographic monitor (BIS monitor) can be useful [28-31]. In this study, BIS and TCI were used to establish the dosage of propofol. This method sets the blood concentration, and administration of anesthetic establishes a constant anesthesia level. Accordingly, administering a lower dose because the blood concentration was set low may decrease BIS if drug metabolism is slow. Thus, if drug metabolism is slow, the administered dose is decreased, and the time to emerge from the same anesthesia level is delayed.

Lidocaine has been reported to reduce propofol requirements during the maintenance phase of total intravenous anesthesia [32]. This report used a large quantity of intravenous lidocaine (>1.5 mg/kg). This report also indicated that lidocaine reduces the propofol requirements, particularly during surgical stimulation, and the blood levels of propofol measured at the end of the infusions were similar to those without lidocaine. Therefore, lidocaine does not influence emergence, even when used in large quantities. In our study, we used a low level of lidocaine (no more than 1.8 ml 2% lidocaine), and the administration route was local, not intravenous. Therefore, we believe that locally administered lidocaine has little effect on propofol anesthesia.

Our study has several limitations. From the present result and other reports, we considered that antiepileptics inhibit propofol metabolism, resulting in elevation of plasma concentrations of propofol. However, we did not examine the blood concentration. Rather, we measured the value that was displayed by the TCI pump when the patient awoke. Determination of propofol metabolism may be possible if the propofol blood concentration at the end of administration and later are measured. Another limitation of our study is that all antiepileptics were grouped together for analysis because not many patients were taking a single antiepileptic alone. As previously stated, all antiepileptics used by patients in this study have an inhibitory effect on propofol metabolism. However, if the blood concentration of the antiepileptic had been measured, the strong influence of the antiepileptic drug on propofol metabolism may have been detectable. Another limitation of our study is the small number of patients except for those with ID. The number with AU with Epi was three patients. In this study, in patients with ID given an antiepileptic, the required dose of propofol was significantly lower, the modeled propofol blood concentration at emergence was significantly lower, and the time to emerge was significantly longer compared to those not given an antiepileptic. Patients with AU and patients with CP did not show a significant difference between use and non-use of an antiepileptic. However, if we had examined a larger number of patients with AU and CP, we may have been able to precisely examine the influence of the antiepileptic drug in each type of disability.

In this study, in patients not given an antiepileptic, we found no significant differences in the dose of propofol administered, time to emerge from intravenous general anesthesia, or modeled propofol blood concentration at the time of emergence among patients with AU, CP, and ID. On the other hand, in patients with ID given an antiepileptic, the required dose of propofol was significantly lower, the modeled propofol blood concentration at emergence was significantly lower, and the time to emerge was significantly longer compared to those not given an antiepileptic.

## Conclusions

The propofol dose required for anesthesia and the time to emerge from anesthesia are not affected by the type of disability but are mainly affected by the use of an antiepileptic.
